# Neuronal Migration Generates New Populations of Neurons That Develop Unique Connections, Physiological Properties and Pathologies

**DOI:** 10.3389/fcell.2019.00059

**Published:** 2019-04-24

**Authors:** Bernd Fritzsch, Karen L. Elliott, Gabriela Pavlinkova, Jeremy S. Duncan, Marlan R. Hansen, Jennifer M. Kersigo

**Affiliations:** ^1^Department of Biology, University of Iowa, Iowa City, IA, United States; ^2^Department of Otolaryngology, University of Iowa, Iowa City, IA, United States; ^3^Institute of Biotechnology ASCR, Vestec, Czechia; ^4^Department of Biological Sciences, Western Michigan University, Kalamazoo, MI, United States

**Keywords:** neuronal migration, differential function, neuronal pathology, neuronal pathfinding, neuronal functionality

## Abstract

Central nervous system neurons become postmitotic when radial glia cells divide to form neuroblasts. Neuroblasts may migrate away from the ventricle radially along glia fibers, in various directions or even across the midline. We present four cases of unusual migration that are variably connected to either pathology or formation of new populations of neurons with new connectivities. One of the best-known cases of radial migration involves granule cells that migrate from the external granule cell layer along radial Bergman glia fibers to become mature internal granule cells. In various medulloblastoma cases this migration does not occur and transforms the external granule cell layer into a rapidly growing tumor. Among the ocular motor neurons is one unique population that undergoes a contralateral migration and uniquely innervates the superior rectus and levator palpebrae muscles. In humans, a mutation of a single gene ubiquitously expressed in all cells, induces innervation defects only in this unique motor neuron population, leading to inability to elevate eyes or upper eyelids. One of the best-known cases for longitudinal migration is the facial branchial motor (FBM) neurons and the overlapping inner ear efferent population. We describe here molecular cues that are needed for the caudal migration of FBM to segregate these motor neurons from the differently migrating inner ear efferent population. Finally, we describe unusual migration of inner ear spiral ganglion neurons that result in aberrant connections with disruption of frequency presentation. Combined, these data identify unique migratory properties of various neuronal populations that allow them to adopt new connections but also sets them up for unique pathologies.

## Introduction

Most central nervous system (CNS) neurons become postmitotic near either the ventricle or the pia mater of the brain, typically through the cell division of a radial glia cell, whereas all peripheral nervous system (PNS) neurons are derived either from neural crest or peripheral placodes (Reiprich and Wegner, 2015; Allen and Lyons, 2018). After exiting the cellcycle, neurons migrate along various trajectories to their final destination to adopt unique fates through unique positions and associated unique connections (Sidman and Rakic, 1973). Mechanistically, migration is an expansion of the internuclear translocation of radial glia precursors and is best understood for radial neuronal migration (Allen and Lyons, 2018). By contrast, the molecular processes governing various tangential migrations are much less understood. Like radial migration, a process is extended and the nucleus translocates within this leading process either along radial glia fibers or criss-crossing through the radial glia fibers (Fritzsch and Northcutt, 1993) possibly interacting with other glia cells, including oligodendrocytes (Allen and Lyons, 2018) or Schwann cells (Bixby et al., 1988; Lilien and Balsamo, 2005). A classical example of such migration along radial glia fibers is the migration of cerebellar granule cells along radial Bergman glia fibers from the external granule layer to the internal granule cells (Horn et al., 2018), leaving rarely a few so called “ectopic granule cells” in the external granule cell layer (Frotscher, 1997). This migration generates about 60% of all neurons in a mammalian brain (Herculano-Houzel, 2009), making the cerebellar granule cells the largest single population of neurons in the mammalian brain.

**FIGURE 1:**
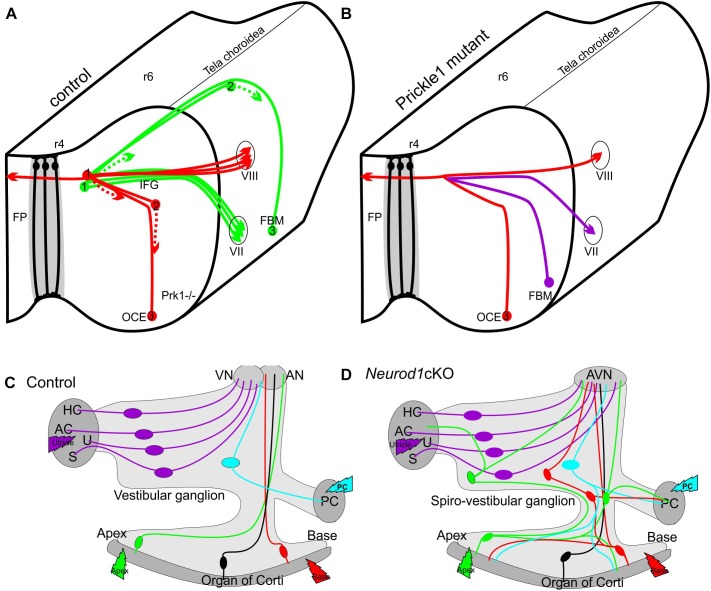
Schematic depiction of migration defects with projection changes in the CNS **(A,B)** and PNS **(C,D)**. Deletion of (Prk1-/-) results in aberrant migration **(B)** of Facial Branchial Motor neurons (FBM) which migrate more like the olivo-cochlear efferents (OCE) in control mice **(A)** instead along the floor plate (FP) from rhombomere 4 (r4) to r6 (A). 1,2,3 indicates different migratory positions, dotted line indicates leading process. Neither crossing of the floor plate by efferents (red fiber in **A,B)** nor peripheral projections seem to be affected. In contrast, in the PNS the unusual migration of some spiral ganglion neurons into the vestibular ganglion (green, red) in Neurod1 cKO mice **(D)** results in aberrant connections of spiral ganglion neurons (green and red cells in **D)** to vestibular sensory epithelia (AC, anterior crista; AN, auditory nerve; AVN, auditory-vestibular nerve; HC, horizontal crista; PC, posterior crista; S, saccule; U, utricle). Modified after (Yang et al., 2014a; Macova et al., 2019).

Depending on the nuclear translocation, the leading process may develop into a dendrite or may transform partially into the axon (Figure 1). For example, facial branchial motor neurons become postmitotic near the floor plate in rhombomere 4 of the hindbrain (Fritzsch and Nichols, 1993). They send out a leading process along the floor plate to rhombomere 6 and eventually translocate the nuclei within this leading process, turning the trailing process into an extension of the axon (Yang et al., 2014a; Glasco et al., 2016; Han et al., 2018; Gruner et al., 2019). Likewise, some ocular motor neurons and some inner ear efferents can translocate within a leading process to the contralateral side, turning the leading process into an extension of the axon as the nucleus and cell bodies translocate across the floor plate (Puelles, 1978; Fritzsch et al., 1995; Simmons et al., 2011; Bjorke et al., 2016; Fritzsch et al., 2017). This process is for unknown reasons restricted to a subset of inner ear efferents in some vertebrates but not in mammals (Simmons et al., 2011). Among vertebrate ocular motor neurons contralateral migration involves only those neurons innervating the superior rectus and levator palpebrae muscles of the orbit (Fritzsch et al., 1990; Naujoks-Manteuffel et al., 1991; Cheng et al., 2014). In addition to intrinsic migration, some cells delaminate from the infolding neural tube to become the uniquely diverse neural crest cell population that gives rise to sensory neurons in ganglia but also a rich variety of non-sensory cells (Lindsley et al., 2007; Dupin et al., 2018). Relative to neural crest migration, inner ear placode derived neuronal migration has been much less investigated (Fritzsch et al., 2002; Yang et al., 2011). Much like neural crest derived neurons, inner ear derived neurons leave the forming otic epithelium to aggregate as a ganglion near the ear (Mao et al., 2014; Goodrich, 2016). However, while neurons connected to the vestibular sensory epithelia translocate within their leading processes to form a ganglion between the ear and the brain, spiral ganglion neurons show less translocation and remain near the sensory epithelium to form a distinct spiral ganglion neuron population within the ear (Yang et al., 2011).

This overview shows that various migration trajectories can generate unique neuronal populations that are separated from other populations with closely associated spatial and temporal origins through their final topology and associated unique input and output properties. While molecular networks of glial cell migration begin to emerge (Jacob et al., 2018; Cantone et al., 2019), in particular, tangential migrations are much less understood at cellular interaction level and the molecular level (Allen and Lyons, 2018; Horn et al., 2018). On a comparative scale, neuronal migration interferes with a simple across species correlation to establish homology of cell populations based on their location and requires the introduction of hodological or connectional analysis to show connectional similarities despite differences in the position of the cell bodies (Fritzsch and Glover, 2007; Fritzsch et al., 2017). Here we will expand on current evidence how those unique migratory properties are affecting their connections and physiology and are involved in certain pathologies.

## Cerebellar Granule Cell Migration

**FIGURE 2:**
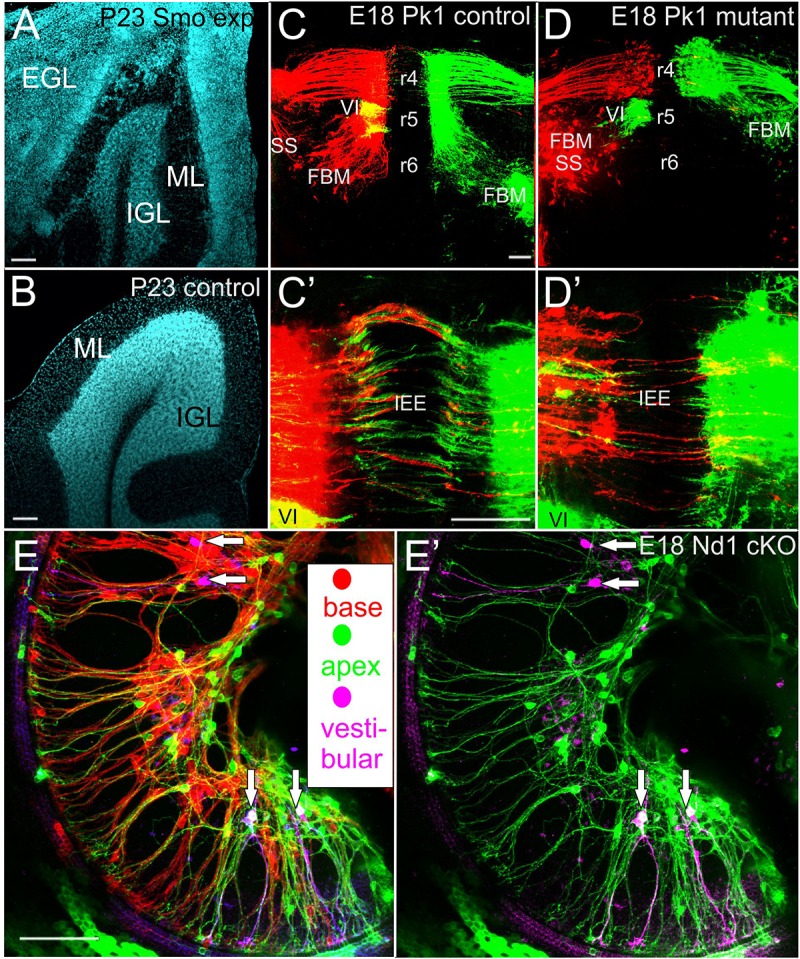
Pathological and connectional defects after differential migration are shown for medulloblastoma **(A,B)**, facial branchial motor neurons **(C,D)** and spiral ganglion neurons **(E)**. The transient, postnatal external granule layer (EGL) is transformed into medulloblastoma after Atoh1-cre mediated conditionally expression of activated smoothened **(A)** reducing the inner granular cell layer (IGL) of lobe IX of the cerebellum compared to the control littermate **(A,B)**. In (Pk1) mutants FBM migration along the floor plate from r4–r6 is abolished. The abducens motor neurons (VI) in r5 show only the color green after retrograde filling **(D)** whereas in control animals VI neurons appear yellow due to the optical overlap of the VI (green) and VII (red). Despite massive effects on longitudinal migration of FBM **(C,D)** the contralateral projection of inner ear efferent (IEE) show only comparatively limited effects **(C’,D’)**. Only spiral ganglion neurons of mice with a conditional deletion of Neurod1 show spiral ganglion neurons with multiple branched processes (vertical arrows in **E,E’)** that also have branches to the vestibular organs (lilac color) in the middle turn. In addition, both apical (green) and basal (red) dye applications label spiral ganglion neurons near the base and apex **(E)**. Removing the red, basal application labeled spiral ganglion neurons reveal that some spiral ganglion neurons project not only to vestibular organs but in addition to the apex **(E’)**. Bar indicates 100 um in all images. Modified after (Yang et al., 2014a; Macova et al., 2019).

Granule cell development and migration have been extensively studied and the past literature has been recently reviewed (Leto et al., 2016; Horn et al., 2018) and molecules guiding that migration have been identified (Driver et al., 2017). Importantly, the external granule layer consists of Atoh1 expressing highly proliferative precursors with a mitotic rate driven mostly by the sonic hedgehog (Shh) signaling pathway (Flora et al., 2009). Atoh1 regulates expression of Neurod1 that is needed for differentiation of granule cells and their migration from the external granular layer (EGL) along Bergman glia fibers to the internal granule cell layer [IGL] using Ttc21b (Driver et al., 2017; Horn et al., 2018). Given that the EGL generates about 60% of all brain neurons (von Bartheld et al., 2016) and that proliferation is indicative for 60% of tumor formation (Tomasetti and Vogelstein, 2015a), it is not surprising that medulloblastoma (MB) formation is one of the more frequent infant tumors (Northcott et al., 2012; Taylor et al., 2012). MB uses the Shh pathway to transform the highly proliferative EGL cells into uncontrolled proliferative tumor cells (Goodrich et al., 1997). Enhancing the Shh pathway signaling through constitutively active Smo selectively activated in Atoh1-cre positive precursors (Matei et al., 2005) reliably results in rapidly growing MB transformation of the cerebellum (Schuller et al., 2008). Given that Atoh1-cre is expressed with delay in different lobes of the cerebellum (Pan et al., 2009), these areas can be studied to verify where transformations are most prominent: EGL neuronal precursors, migratory granule cells or granule cells differentiated in the IGL. Without a doubt, *Atoh1-cre*-induced Smo preferentially transforms the EGL into a rapidly growing MB (Figure 2A,B). Pre-migratory neuroblasts that undergo rapid division, a main cause for carcinogenic transformation (Tomasetti and Vogelstein, 2015a,b), are thus the primary source of MB formation. While much of the current approaches to treat MB focus on Shh antagonists such as vismodegib (Morrissy et al., 2017; Zarei et al., 2017; Tan et al., 2018), the insight that starting points of MB are pre-migratory granule cell precursors suggest a possible additional approach: induce premature migration that may deplete EGL and truncate the numbers of granule cells, which should certainly reduce tumor progression. Indeed, using an induced overexpressing system of Neurod1, we recently obtained evidence that certain late developing MB forms can be slowed down.

## Oculomotor Neuron Migration

The oculomotor complex of vertebrates consists of motor neurons that form three distinct cell groups for cranial nerves (CNIII, IV, VI) with distinct patterns of innervation that are partially conserved across vertebrates (Fritzsch et al., 1990). These motor neurons have been designated for over 100 years as somatic motor neurons, implying serial homology to spinal somatic motor neurons. However, recent molecular insights have led to a reclassification of these motor neurons, in particular, the oculomotor (CNIII) and trochlearis (CNIV) motor neurons, as special somatic motor neurons (Fritzsch et al., 2017). These motor neuron populations have a critical dependence on the midbrain/hindbrain boundary signaling cascade (Glover et al., 2018). During vertebrate development there is one subpopulation of CNIII innervating the superior rectus and levator palpebrae muscles (only in mammals) that migrates across the midbrain floor plate (Puelles, 1978; Fritzsch and Nichols, 1993; Fritzsch et al., 1995). The molecular features guiding this contralateral migration such as Slit/Robo are beginning to emerge (Bjorke et al., 2016) as well as other unusual features of this population. One aspect we explore here concerns the correlation between the unusual migration and their target navigation in the orbit.

The fibers to the superior rectus division of CNIII branch upon entry into the orbit, indicating that the migration and branching pattern are two unique aspects specific to this population of motor neurons. This is important as this branching was recently shown to explain a human eye movement defect, congenital fibrosis of the extraocular muscles type 1 (CFEOM1). Specifically, it was found that a mutation in a human tubulin associated transporter, Kif21a, causes stalling of oculomotor nerve fiber axons destined to reach the superior rectus and levator palpebrae. In this disease since the muscle fibers are not innervated, they turn into connective tissue in humans as well as in a mouse model of this human disease (Cheng et al., 2014). In addition, because the nerve fibers do not reach the correct peripheral target to supply trophic support, this motor neuron population is specifically reduced through apoptosis, as the neural soma are translocating contralaterally (Cheng et al., 2014). Since this population of contralaterally migrating oculomotor motor neurons is the only motor neuron population in mammals doing so, it is affected by a molecule that is ubiquitously expressed. What remains to be shown is the identity of the unique cargo of Kif21a that initiates the premature branching of the contralateral nerve fibers in this unusual gain of function human disease.

## Facial Branchial Motor Neurons and Inner Ear Efferents

Facial branchial motor (FBM) neurons are motor neurons (Glover et al., 2018) that innervate all muscles of facial expression and are known for acute diseases (Han et al., 2018), such as complete facial paralysis that directly involves the motor neurons (Bell’s palsy, lower motor weakness) or partial facial paralysis, sparing the frontalis and orbicularis oculi muscles (upper motor neurons after infarct of the motor cortex). Due to the bilateral innervation of the facial motor neurons innervating the frontal and orbicularis oculi muscles by the motor cortex, unilateral cortical infarct spares the ability to wrinkle the forehead and close the eyes, which serves as a clinical diagnostic feature to distinguish between upper (cortical) and lower (hindbrain) facial motor neuron diseases. Details of molecular guidance begin to emerge and reveal that this is mostly associated with planar cell polarity signals such as Vangl2, Prickle1, Clsr1 (Glasco et al., 2016; Han et al., 2018; Yang et al., 2014a). It has been argued that correct FBM migration is a prerequisite for this unusual bilateral/unilateral innervation (Yang et al., 2014a). Unfortunately, all mouse mutants with FBM neuron migration defects are not viable (Müller et al., 2003; Yang et al., 2014a; Glasco et al., 2016; Gruner et al., 2019) thus precluding to test the possibility that the unusual cortical innervation patterns of FBM neurons require the correct migration. It should also be noted that FBM neuron migration is extremely variable among vertebrates, ranging from no longitudinal migration at all (frogs, lampreys) to migration into the cervical spinal cord (some lungless salamanders) and is possibly correlated in all these cases with unique patterns of innervation for movement control (Fritzsch, 1998; Murakami et al., 2005). Obviously, the evolutionary novelty of facial muscles of expression of the mammalian type correlates in all mammals with a caudal migration of the FBM neurons along the floor plate from rhombomere 4 to rhombomere 6 (Gruner et al., 2019). However, a similar caudal migration by two rhombomeres is found in other vertebrates such as some bony fish (Yang et al., 2014a), indicating that the migration is not ubiquitously linked with the appearance of muscles of facial expression but maybe linked to the cortical control of these motor neurons in mammals.

Other than various paralyzes outlined above, facial motor neurons appear to be affected in one rare congenital disease, branchio oculo facial syndrome (BOFS^1^). Mutations in transcription factor AP2-alpha [TFAP2A] are underlying this variably penetrant, dominantly inherited syndrome (Kousa et al., 2019). This gene codes for a retinoic acid-inducible activator of transcription that affects multiple tissues, including the differentiation of neural tube and palate formation in a mouse model of TFAP2A mutation (Kousa et al., 2019). This gene is widely expressed in the CNS, PNS, and ocular tissue but may more indirectly affect FBM neuron migration based on the well-known effects of RA on various developmental tissues, including specification of rhombomere identity (Manns and Fritzsch, 1992; Parker et al., 2016). The facial branchial motor neurons of *Tfap2a* mouse mutant (Kousa et al., 2019) should be investigated given that certain PCP genes have both defects of the palate (Yang et al., 2014b), and show FBM migration defects (Yang et al., 2014a). Cleft palate is one major feature of *Tfap2a* mutant mice (Kousa et al., 2019) but no data exist on FBM migration in this mutant.

Inner ear efferent neurons are found in all vertebrates, either associated with the FBM neurons or in a distinct location through differential migration (Simmons et al., 2011; Fritzsch and Elliott, 2017). One of the genes suggested to provide the unique identity, *Gata3* (Karis et al., 2001), is also involved in partial DiGeorge syndrome (hypoparathyroidism, deafness, and renal malformations; HDR^2^). Unfortunately, identifying the unique function of Gata3 in inner efferent guidance and migration requires unique deletion only in the inner ear efferents as Gata3 is also essential for cochlear development (Duncan and Fritzsch, 2013). The cochlea is the most derived target of inner ear efferents that has evolved a unique motor to enhance minute cochlear vibrations needed for sound amplification (Dallos et al., 2008; Elliott et al., 2018). One molecularly underexplored separation is the segregation of different sub-populations of neurons of the olivo-cochlear efferent system. Only olivo-cochlear efferent neurons that directly innervate the contractile outer hair cells are bilaterally distributed, whereas those efferents that innervate the afferent peripheral processes of spiral ganglion neurons near inner hair cells are nearly exclusively ipsilateral (Simmons et al., 2011). In addition, those innervating spiral ganglion neuron afferent processes are located in a different brainstem nucleus compared to those ending on outer hair cells, again pointing out that different peripheral projection patterns correlate with different perikaryon localization and implying that differential migration also affects pathway selection. Comparable to the *Kif21a* mutation exclusively affecting one population of contralaterally migrating ocular motor neurons, pathfinding properties of inner ear efferents and certain other neuronal populations are selectively affected by mutations in Nova proteins that regulate differential splicing (Saito et al., 2016). Below we will explore this correlation further using recent data on the differential migration of inner ear sensory neurons.

## Inner Ear Sensory Neurons: Differential Migration Correlates With Differential Projection of Vestibular and Spiral Ganglion Neurons

The inner ear develops out of a dorso-lateral placode into a complex labyrinth of ducts and recesses with six sensory epithelia and two distinct ganglia (Fritzsch et al., 2002; Yang et al., 2011). Vestibular ganglion neurons translocate within the central process close to the brain, whereas spiral ganglion neurons remain within the coiled cochlea and project centrally to the cochlear nucleus (Fritzsch et al., 2015). As in the examples above, there is an apparent correlation with differential migration and distinct projection patterns (Figure 1, 2). This correlation has been explored in several papers for cell-autonomous and non-cell-autonomous dependency. While none of the effects found thus far can be related to a known human pathology, it is clear that at least in one viable case, altered translocation correlates with miss-wiring and altered function (Macova et al., 2019).

Translocation of spiral ganglion neurons is, like translocation within the brain, correlated with specific interactions with glial cells, neural crest derived Schwann cells. Eliminating Schwann cells using a targeted deletion of the essential Schwann cell differentiation factors *ErbB2* (Morris et al., 2006) or *Sox10* (Mao et al., 2014) results in a disorganized innervation of the organ of Corti with overshooting fibers and a translocation of most spiral ganglion neurons toward the brain to overlap with vestibular ganglion neurons. Interestingly enough, the central processes, within which the translocation takes place, seemingly projects in a normal fashion to the cochlear nuclei, whereas the peripheral processes are misguided and many bypass their peripheral target (hair cells) in the organ of Corti. The remaining innervation of the organ of Corti shows a chaotic pattern (Mao et al., 2014). Unfortunately, even conditional mutants involving loss of Schwann cells are not viable and thus, the physiological effect of the differential guidance has thus far not been explored in these mutants.

A somewhat similar phenotype of overshooting translocation of spiral ganglion neurons has been reported in a conditional deletion model of the transcription factor Neurod1 (Jahan et al., 2010). Unfortunately, due to a very early conditional deletion of *Neurod1* in this specific model, only few neurons survive making it impossible to distinguish between a general viability defect and a specific effect of *Neurod1* on migration and target homing. This problem was recently overcome using a later expressing cre line that results in viable mice that can be studied for the physiological impact of loss of *Neurod1* (Macova et al., 2019). In Isl1-cre mediated *Neurod1* deletion, surviving spiral ganglion neurons migrate, to a variable extent, away from the cochlea to overlap with vestibular ganglion neurons to generate a new ganglion, the spiro-vestibular ganglion (Figure 1). Thanks to a delayed loss of *Neurod1*, these mice retain many of the spiral ganglion neurons. Detailed analysis revealed that it is not only the migration that is disrupted but both the central and peripheral projections are disorganized. Many spiral ganglion neurons develop a premature fiber growth to the outer hair cell compartment that shows in many areas processes that overshoot the organ of Corti, much like in conditional deletion of *Sox10* (Mao et al., 2014). In contrast to Schwann cell mutants with a somewhat similar dislocation of spiral ganglion neurons, conditional deletion of *Neurod1* shows variably penentrant central projection errors. The early deletion of *Neurod1* using Pax2-cre results in a mixed “cochleo-vestibular” nerve with mostly overlapping projections to both the cochlear and vestibular nuclei (Jahan et al., 2010). The somewhat later deletion of *Neurod1* using Isl1-cre shows many more surviving spiral ganglion neurons in various positions inside and outside the ear and displays a nearly completely overlapping cochleotopic projection to the cochlear nuclei (Macova et al., 2019).

Since cochleotopic connection between the ear and auditory nuclei is the connectional substrate for tonotopic (frequency specific) hearing, physiological tests can reveal the effect of disorganized innervation on frequency specific hearing by tuning curves. In principle, one of two outcomes were expected: the topographical wiring will be corrected for by the frequency specific filtering mechanisms or the miswiring of the cochlea onto cochlear nuclei results in aberrant tuning curves. Clearly, the tuning properties of central auditory neurons are abnormal and show in most cases only weak if any tuning properties at all. Combined these data suggest that migration and pathfinding in spiral ganglion neurons depend on Neurod1 that regulates the molecular basis of Schwann cell interaction both to read the stop signal and to navigate in a topographical specific way to establish the connectional substrate for tonotopic, frequency specific hearing. Given the complexity of the mouse mutants needed to have viable mice with the specific targeted deletion of *Neurod1*, a transcription factor that is widely expressed in the PNS, the CNS, the pancreas (beta-cells) and the intestine (enteroendocrine cells), it seems very unlikely that a human pathology will ever be encountered matching this specific phenotype. Nevertheless, the resulting mouse pathology is extremely informative to reveal the molecular basis of frequency specific hearing and the limitations of physiological refinement of frequency specific sound perception as specific case of sensory map formation (Fritzsch et al., 2019).

In summary, we present four cases of migration related CNS and PNS pathologies that correlate specific migrations with defined pathologies or connection details of distinctly migrating populations. While much of the presented data are at the level of correlation, at least one case shows that migration, fiber guidance and overall wiring critically depends on a single transcription factor that also plays a crucial role in one of the other cases of neuronal migration, Neurod1. It remains to be shown, how other transcription factor(s) affect the differential migration and projection of the two other populations presented here, and what is the molecular nature of cellular interactions with surrounding glia fibers that direct the process growth and direction and level of translocation.

## Author Contributions

BF conceived the review. KE, GP, JD, and JK contributed the images and parts of writing. KE, GP, JD, MH, and JK reviewed and edited the manuscript.

## Conflict of Interest Statement

The authors declare that the research was conducted in the absence of any commercial or financial relationships that could be construed as a potential conflict of interest.

## Footnotes

1 https://omim.org/entry/113620

2 https://omim.org/entry/146255?search=gata3&highlight=gata3
